# Annual estimates of the burden of seasonal influenza in the United States: A tool for strengthening influenza surveillance and preparedness

**DOI:** 10.1111/irv.12486

**Published:** 2018-02-14

**Authors:** Melissa A. Rolfes, Ivo M. Foppa, Shikha Garg, Brendan Flannery, Lynnette Brammer, James A. Singleton, Erin Burns, Daniel Jernigan, Sonja J. Olsen, Joseph Bresee, Carrie Reed

**Affiliations:** ^1^ Influenza Division National Center for Immunization and Respiratory Diseases Centers for Disease Control and Prevention Atlanta GA USA; ^2^ Battelle Memorial Institute Atlanta GA USA; ^3^ Immunization Services Division National Center for Immunization and Respiratory Diseases Centers for Disease Control and Prevention Atlanta GA USA

**Keywords:** burden, influenza, United States

## Abstract

**Background:**

Estimates of influenza disease burden are broadly useful for public health, helping national and local authorities monitor epidemiologic trends, plan and allocate resources, and promote influenza vaccination. Historically, estimates of the burden of seasonal influenza in the United States, focused mainly on influenza‐related mortality and hospitalization, were generated every few years. Since the 2010‐2011 influenza season, annual US influenza burden estimates have been generated and expanded to include estimates of influenza‐related outpatient medical visits and symptomatic illness in the community.

**Methods:**

We used routinely collected surveillance data, outbreak field investigations, and proportions of people seeking health care from survey results to estimate the number of illnesses, medical visits, hospitalizations, and deaths due to influenza during six influenza seasons (2010‐2011 through 2015‐2016).

**Results:**

We estimate that the number of influenza‐related illnesses that have occurred during influenza season has ranged from 9.2 million to 35.6 million, including 140 000 to 710 000 influenza‐related hospitalizations.

**Discussion:**

These annual efforts have strengthened public health communications products and supported timely assessment of the impact of vaccination through estimates of illness and hospitalizations averted. Additionally, annual estimates of influenza burden have highlighted areas where disease surveillance needs improvement to better support public health decision making for seasonal influenza epidemics as well as future pandemics.

## 1 INTRODUCTION

Estimates of the burden of seasonal influenza are broadly useful for public health, helping national and local authorities monitor epidemiologic trends, plan and allocate resources, demonstrate the impact of vaccine programs as well as other public health and clinical interventions, and inform the public, clinicians, and policymakers about the importance of influenza and influenza prevention.

Estimates of the burden of seasonal influenza in the United States have evolved over time. First, estimates focused on the number of deaths due to influenza in the 1960s.^1^ With better access to hospital records, estimates were expanded to include influenza‐related hospitalizations.^2^, ^3^ During the 2009 H1N1 pandemic, there was a need to describe the burden of less severe outcomes, which further expanded the burden estimation to include outpatient medical visits and illness in the community.^4^

Changes were also made to the methods used to generate estimate of influenza burden. Statistical models were initially used to estimate excess deaths and hospitalizations, those that occur above what is predicted based on historical trends.^1^, ^5^, ^6^ During the 2009 H1N1 pandemic, there was a move toward using a multiplier that could extrapolate rates of hospitalization to rates of less severe disease.

Historically, new estimates of influenza‐related mortality or hospitalization over multiple influenza seasons were published periodically, as new data became available.^5^, ^6^, ^7^ However, a hallmark of influenza is its variability from one season to the next and periodic assessments of the burden of influenza fail to capture the full extent of seasonal variation. For this reason, the Centers for Disease Control and Prevention (CDC) has transitioned from providing periodic estimates to reporting annual estimates of influenza burden in the United States. Annual estimates of disease burden, in combination with annual assessments of influenza vaccine coverage and vaccine effectiveness in preventing disease, allow for timely evaluation of influenza prevention and control efforts.

## 2 GENERATING ANNUAL ESTIMATES OF INFLUENZA BURDEN

Data from the 2010‐2011 through 2015‐2016 influenza seasons (October through April) have been used on an annual basis to estimate the burden of seasonal influenza and the disease burden averted by influenza vaccination in the United States.^8^, ^9^, ^10^, ^11^, ^12^, ^13^ The methods have previously been described in detail (Fig. S1).^7^, ^9^, ^11^, ^12^, ^13^, ^14^

Briefly, rates of hospitalization with laboratory‐confirmed influenza were obtained from the Influenza Hospitalization Surveillance Network (FluSurv‐NET), a population‐based surveillance conducted in 14 geographically distributed states.^15^ Hospitalization rates were generated by age group (0‐4, 5‐17, 18‐49, 50‐64, and ≥65 years). Rates were adjusted for influenza testing practices and test sensitivity and then applied to the US population to obtain estimates of the number of influenza‐associated hospitalizations that occurred each season.

Estimates of excess deaths related to influenza were based on a statistical model of the weekly number of deaths obtained from the National Center for Health Statistics.^16^ The model accounts for seasonal trends in mortality and weekly circulation of influenza and respiratory syncytial virus, obtained from national virologic surveillance.^16^, ^17^ The model was fitted using Markov chain Monte Carlo methods, yielding “point estimates” (mean or median of the empirical posterior distribution) and “confidence intervals” (95% credible intervals) for the number of deaths attributable to influenza. Data on deaths with pneumonia or influenza listed as a cause of death were used in the statistical model because they are available in near real time. However, most influenza‐related deaths are likely not due directly to influenza virus infection but may be due to secondary bacterial infection or worsening of underlying chronic health conditions, such as chronic heart or lung disease. Even when influenza likely contributed to the events leading to a death, it may not be recognized and is rarely listed on the death certificate. From prior analyses, the number of deaths associated with influenza may be two to four times higher than the number of deaths related to influenza that have pneumonia or influenza listed on the death certificate.^7^, ^18^ Deaths with any respiratory or circulatory causes listed on the death certificate are likely more inclusive of deaths related to influenza than deaths with pneumonia or influenza causes; therefore, additional statistical models were created using death from respiratory or circulatory causes. Data on respiratory and circulatory deaths were available with a 3 year lag; therefore, in 2016, data were available and summarized for the 2010‐2011 season through the 2013‐2014 season only.

Estimates of the case‐to‐hospitalization ratio, obtained from studies during the 2009 pandemic in the United States,^4^ were used to calculate the number of illness episodes that occurred in the community from the number of hospitalizations with laboratory‐confirmed influenza. Estimates of the proportion of ill persons who sought medical care, obtained from a nationwide behavior survey conducted in the United States during the 2009 pandemic,^19^ were used to estimate the number of outpatient medical visits relative to the estimated number of influenza illnesses in the community.

Estimates of the numbers of influenza‐associated illnesses, outpatient medical visits, hospitalizations, and deaths prevented by influenza vaccination were derived from burden estimates, influenza vaccination coverage, and vaccine effectiveness, as previously described.^9^ Briefly, estimates of monthly influenza vaccine coverage by age group (6 months‐4 years, 5‐17, 18‐49, 50‐64, and ≥65 years) and annual vaccine effectiveness estimates were used to estimate the number of influenza outcomes that would have occurred in the absence of vaccination, assuming equal vaccine effectiveness against each outcome.^20^, ^21^ From the estimates of burden estimates, vaccination coverage, and vaccine effectiveness, we calculated age group‐specific hypothetical numbers of illness, medical visits, hospitalizations, and respiratory or circulatory deaths related to influenza per month that would have occurred in the absence of vaccination (assuming only a direct vaccination effect). The outcomes prevented by vaccination were the difference between these hypothetical numbers and the burden estimates for the actual population. The fraction of hospitalizations prevented by vaccination was the total number of hospitalizations averted divided by the number of hospitalizations that would have occurred in the absence of vaccination.

The model of averted outcomes was also used to estimate the incremental benefits expected from increasing vaccine coverage or vaccine effectiveness. The number needed to vaccinate to prevent one influenza‐associated illness was estimated by dividing the size of the vaccinated population in the United States by the number of illnesses prevented by vaccination.

Estimates of the number of illnesses, outpatient medical visits, hospitalizations, and respiratory and circulatory deaths related to seasonal influenza in the United States and the number of illnesses, medical visits, hospitalizations, and deaths related to influenza that were prevented by influenza vaccination are updated annually and published online.^13^

## 3 ESTIMATED BURDEN OF INFLUENZA‐ASSOCIATED ILLNESS, OUTPATIENT MEDICAL VISITS, HOSPITALIZATIONS, AND DEATHS

**Figure 1:**
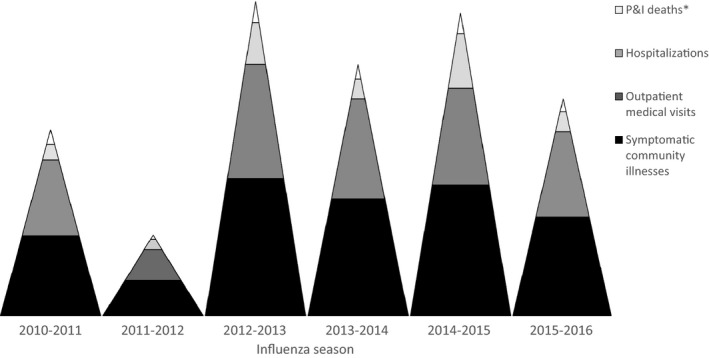
Relative number of community illnesses, outpatient medical visits, hospitalizations, and deaths associated with seasonal influenza—United States, 2010‐2011 through 2015‐2016 influenza seasons. *Only data on pneumonia and influenza deaths were available in real time during an influenza season; however, pneumonia and influenza deaths are only a subset of the total deaths associated with influenza that occur each year, which may be 2 to 4 times higher when other complications are also considered

Over the past six influenza seasons in the United States (2010‐2011 through 2015‐2016), we estimate that influenza‐associated illnesses have ranged from a low of 9.2 million to a high of 35.6 million illnesses, with variation by age (Table 1). Outpatient medical visits related to influenza have ranged from 4.3 million to 16.7 million, while influenza‐associated hospitalizations have ranged from 139 000 to 708 000. From 2010‐2011 through 2013‐2014 influenza seasons, influenza‐associated respiratory and circulatory deaths have ranged from a low of 12 000 to a high of 56 000, and associated pneumonia and influenza deaths have ranged from 4000 to 12 000 over the six seasons from 2010‐2011 through 2015‐2016. Figure 1 is a visual representation of the burden of influenza, which depicts the relative magnitude of each season's burden in the United States.

**Table 1:** Six‐season range of symptomatic community illnesses, outpatient medical visits, hospitalizations, and excess deaths related to influenza, by age group—United States, 2010‐2011 through 2015‐2016 influenza seasons

Age group	Symptomatic community illness	Outpatient medical visits	Hospitalizations	Excess deaths	
				Pneumonia & influenza^a^	Respiratory & circulatory^b^
Overall	9 200 000–35 600 000	4 200 000–16 700 000	139 000–708 000	4000–20 000	12 000–56 000
<5 y	900 000–3 800 000	600 000–2 500 000	6000–26 000	60–300	100–700
5–17 y	1 900 000–6 900 000	1 000 000–3 600 000	5000–19 000	50–300	100–600
18–49 y	3 400 000–12 600 000	1 200 000–4 700 000	19 000–71 000	300–2100	900–3600
50–64 y	1 800 000–8 800 000	800 000–3 800 000	20 000–93 000	600–3400	1800–7500
≥65 y	900 000–5 800 000	500 000–3 300 000	87 000–523 000	3000–17 000	9000–43 000

a Only data on pneumonia and influenza deaths were available in real time during an influenza season; however, pneumonia and influenza deaths are only a subset of the total deaths associated with influenza that occur each year, which may be 2 to 4 times higher when other complications are also considered.

b Data on respiratory and circulatory deaths are available with a three‐year lag; therefore, estimates of excess respiratory and circulatory deaths are only available through 2013‐2014 influenza season at this time.

## 4 ESTIMATED INFLUENZA‐ASSOCIATED DISEASE BURDEN PREVENTED THROUGH INFLUENZA VACCINATION

Given the estimates of seasonal incidence of influenza, the associated burden of severe disease, and estimates of influenza vaccine effectiveness and coverage in the United States, we estimate that influenza vaccination has prevented between 1.6 million and 6.7 million illnesses, 790 000‐3.1 million outpatient medical visits, 39 000‐87 000 hospitalizations, and 3000‐10 000 respiratory and circulatory deaths related to influenza each season (Table 2).

**Table 2:** Estimated number and fraction of influenza illnesses, medical visits, hospitalizations, and pneumonia and influenza deaths averted by vaccination, by season—United States, 2010‐2011 through 2015‐2016 influenza seasons^13^

Season	Averted illnesses		Averted medical visits		Averted hospitalizations			Averted deaths			
								Pneumonia and influenza deaths^a^		Respiratory and circulatory deaths^b^	
	No.	95% CI	No.	95% CI	No.	95% CI	Fraction prevented (%)^c^	No.	95% CI	No.	95% CI
2010–2011	5 039 277	3 435 322–7 716 921	2 514 353	1 702 599–3 885 779	70 821	33 965–141 708	20.8	3434	1422–6906	9880	3883–19 362
2011–2012	1 981 571	1 160 279–3 666 130	968 312	555 687–1 809 753	39 301	17 610–88 885	22.7	1227	505–2450	3618	1400–6909
2012–2013	5 628 332	4 235 767–8 327 082	2 701 875	1 997 056–4 085 452	61 522	31 580–162 836	11.1	1823	724–5517	5280	2149–15 029
2013–2014	6 683 929	5 037 991–8 898 309	3 080 284	2 252 594–4 190 948	86 730	56 447–129 736	21.5	3840	2298–5844	9172	5267–14 465
2014–2015	1 606 813	609 744–3 456 741	792 958	296 449–1 744 001	47 449	10 795–144 291	7.5	1419	312–4255	–	–
2015–2016	5 083 498	3 538 000–7 081 344	2 504 323	1 725 971–3 532 835	71 479	42 344–112 228	18.9	2882	1588–4562	–	–

a Only data on pneumonia and influenza deaths were available in real time during an influenza season; however, pneumonia and influenza deaths are only a subset of the total deaths associated with influenza that occur each year, which may be 2 to 4 times higher when other complications are also considered.

b Data on respiratory and circulatory deaths are available with a three‐year lag; therefore, estimates on averted respiratory and circulatory deaths are only available through 2013‐2014 influenza season at this time.

c The estimated fraction of influenza‐associated hospitalizations prevented by vaccination was estimated by dividing the estimated number of averted hospitalizations by the estimated number of observed hospitalizations in a given season. Because the estimated number of illnesses in the community and outpatient medical visits is proportional to the estimated hospitalizations, the estimated fraction of community illnesses and outpatient medical visits prevented by vaccination is identical to the fraction of hospitalizations prevented by vaccination.

Surveys in the US population suggest that overall vaccination coverage was 42%‐47% over the past six influenza seasons, although coverage varies considerably by age.^22^ Increases in vaccination coverage can translate to large reductions in influenza‐associated disease burden when influenza viruses in the vaccine are similar to circulating viruses. For example, during the 2015‐2016 influenza season, overall vaccination coverage was 46% and we estimate that vaccination prevented more than 5 million illnesses and 71 000 hospitalizations. If vaccination coverage had been increased by five percentage points across all age groups, more than 500 000 additional illnesses and 6000 additional hospitalizations would have been prevented.

The burden models can also serve to assess the impact of changes in vaccine effectiveness on an annual basis. For example, during the 2014‐2015 influenza season, the influenza A/H3N2 virus that circulated widely drifted, both genetically and antigenically, after vaccine virus recommendations were made, leading to reduced overall vaccine effectiveness (19%) against influenza A viruses.^23^ Given the reduced vaccine effectiveness and high burden of illness, we estimated that, on average, 92 people needed to be vaccinated to prevent a single case of influenza and 3115 people needed to be vaccinated to prevent a single hospitalization. Changes in the vaccine composition and improved vaccine effectiveness (47%) against the circulating influenza virus types during the 2015‐2016 season, along with fewer hospitalized patients, meant that only 29 people needed to be vaccinated, on average, to prevent a single case of influenza and 2033 needed to be vaccinated to prevent one influenza‐associated hospitalization.^21^, ^24^

## 5 DISCUSSION

Periodic estimates of the burden of seasonal influenza in the United States have been made for more than 50 years, focused mainly on mortality and hospitalization related to influenza. In response to the 2009 pandemic and the need for timely data on burden and severity, CDC now generates annual burden estimates and has also expanded burden estimates to include less severe outcomes. These estimates have allowed for timely communication about the importance of vaccination to prevent influenza and helped frame discussions about influenza program goals with policymakers. Annual estimates have reinforced the message that influenza is ever‐changing and will likely differ from one season to the next and we use a range to describe burden in order to more accurately reflect the annual variability of influenza.

In addition to being reported annually, CDC estimates of influenza disease burden were expanded to include outpatient medical visits and symptomatic community illness. Estimates focused on influenza‐related mortality and hospitalizations reinforce the potentially serious nature of influenza, but are a small fraction of the total burden of influenza and can be biased, as they are highly influenced by patterns and policies for hospital admission, influenza testing, and reporting.^11^ On the other hand, estimates of the number of symptomatic community illnesses, for which medical care is not sought but may still result in missed school or work, and outpatient medical visits due to influenza underscore the frequency of influenza illness and its widespread societal impact. We estimate that for every influenza‐related hospitalization, between 11 and 365 more non‐hospitalized cases occur in the community, depending on the age group.^4^, ^8^

Generating annual estimates of influenza burden helped CDC recognize gaps in influenza surveillance activities. For example, there were no means to directly estimate medically‐attended and community illness during the 2009 pandemic. Instead, these portions of influenza burden were indirectly estimated using the rates of influenza‐associated hospitalization and field‐validated multipliers of healthcare utilization and case‐to‐hospitalization ratios from the 2009 H1N1 pandemic. In an effort to fill these gaps, there are now several ongoing efforts and collaborations to gather data that can directly estimate the burden of medically‐attended illness related to influenza as well as symptomatic community illness on a routine basis.^25^, ^26^, ^27^ Not only are these improvements to surveillance helpful during seasonal epidemics, but the creation and optimization of surveillance activities that are routine, robust, and near real time will be helpful when a pandemic occurs.^16^, ^28^

The methods and estimates of seasonal influenza burden are not without limitations. First, influenza vaccination coverage estimates and the multipliers used for estimating outpatient medical visits and symptomatic community illness were derived from survey respondents and are subject to recall bias and non‐response bias. Second, the model of disease prevented through vaccination only calculated outcomes averted through direct protection of persons who were vaccinated, and not indirect or herd protection. Third, this same model assumed a single estimate of vaccine effectiveness against all outcomes and constant effectiveness over the course of the season within each age group, which may be oversimplifications. Fourth, estimates of influenza‐associated mortality were based on an ecologic analysis at the national level and may wrongly attribute mortality above an imputed “baseline” to influenza. In addition, it may not reflect the burden on a different level, such as the state or local level.

Despite their limitations, the models we use for estimating the burden of seasonal influenza are simple and provide timely information that is valuable for public health activities. Burden estimates are invaluable for estimating the economic and societal costs of influenza and making decisions about procurement of vaccines and influenza antivirals before the influenza season begins. At CDC, we have also found that providing estimates of burden on an annual basis has served to strengthen existing influenza surveillance activities, allowed for timely communication of the value of vaccination, improved our understanding of the epidemiology of seasonal influenza in the United States, and enhanced preparedness for future influenza pandemics.
