# Supplementary figures and images for: Annual estimates of the burden of seasonal influenza in the United States: A tool for strengthening influenza surveillance and preparedness
Source: Influenza Other Respir Viruses. 2018 Feb 14;12(1):132–7. doi: 10.1111/irv.12486 (PMC5818346; doi:10.1111/irv.12486)

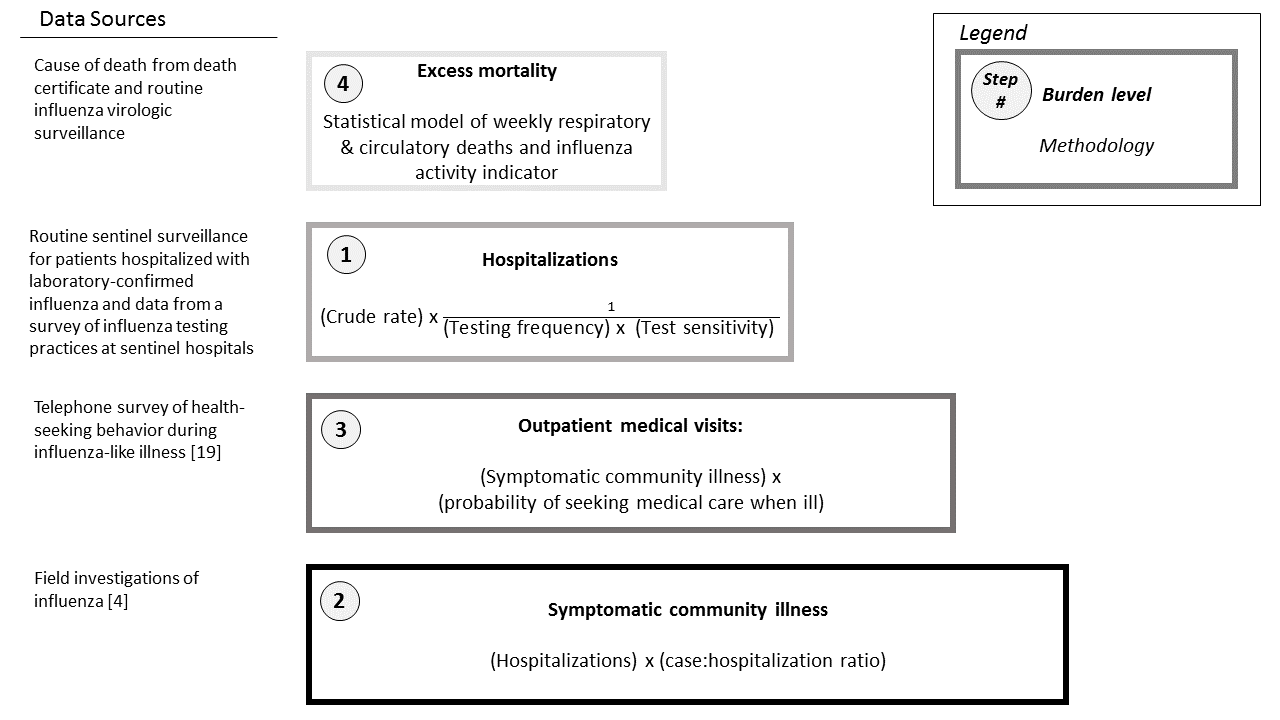

Supplement: Supplementary file 1 [file IRV-12-132-s001.png]
